# The complete mitochondrial genome of the *Pseudecheneis immaculatus* (Siluriformes：Sisoridae)

**DOI:** 10.1080/23802359.2019.1667281

**Published:** 2019-09-19

**Authors:** Tingbing Zhu, Yongfeng He, Deguo Yang

**Affiliations:** Key Laboratory of Freshwater Biodiversity Conservation, Ministry of Agriculture and Rural Affairs of China, Yangtze River Fisheries Research Institute, Chinese Academy of Fishery Sciences, Wuhan, Hubei, China

**Keywords:** *Pseudecheneis immaculatus;* mitochondrial genome, phylogenetic analysis

## Abstract

*Pseudecheneis immaculatus* is an endemic fish species in the Lantsang River. In this study, the complete mitochondrial genome sequence of *P. immaculatus* was determined and analyzed. The 16,432 bp long circular molecule consisted of 13 protein-coding genes, 2 rRNA genes, 22 tRNA genes, and a control region, and all genes show the typical gene arrangement conforming to the vertebrate consensus. The phylogenetic tree showed that *P. immaculatus* is closely related to *P. sulcata*. The present study can provide population genetics information for further exploration of the taxonomic status of *P. immaculatus*.

The Lantsang River (upper Mekong basin) is originated in the Qinghai-Tibet Plateau and flows through Yunnan Province in China. So far, there were only three species of Sisoridae fishes distributed in the upper reaches of Lantsang river basin (Chen and Cao 2000). *Pseudecheneis immaculatus*, a small-sized benthic fish which mainly distributes in the Lantsang River and their tributaries in China. It lives in areas with clear, fast-flowing water and lots of large stones (Wu and Wu 1992). Due to the geological disaster, habitat degradation, exotic species invasion and overfishing in recent years, the natural resources of these species were sharply decreased. Five Sisoridae species have assessed as Endangered by Red List of China’s Vertebrates (Jiang et al. 2016).

In this study, specimen of *P. immaculatus* was collected from the Lantsang River (N 29°06′43.41″, E 98°37′4.95″) at an altitude of 2302 meters in 2018. The specimen was stored in 95% ethanol and kept in the herbarium of Yangtze River Fisheries Research Institute (accession number LC20180410001). The total genomic DNA was extracted from the pelvic fin by a traditional phenol-chloroform method.

The complete mitochondrial genome (GenBank accession number MN082047) was a circular molecule with a length of 16,432 bp, includes 13 protein-coding, 2 ribosomal RNA (rRNA), and 22 transfer RNA (tRNA) genes, and 1 control region (D-Loop). Among the 13 protein-coding genes, 12PCGs (ND1, ND2, ND3, CO2, ATP8, ATP6, CO3, ND4L, ND4, ND5, CytB, ND6) shared start codon ‘ATG’, except for CO1 (start codon ‘GTG’). The single non-coding control region (D-Loop) is 919 bp in length. The two rRNA genes (12S rRNA, 16S rRNA) were 950 bp and 1659 bp in length respectively. The overall base composition of the heavy strain was A (32.1%), T (26.4%), C (26.6%), G (14. 9%), and the content of A + T in the complete genome was 58.5%. Most of the genes were encoded on the heavy strand. Only ND6 and 8 tRNA [tRNA–Gln, –Ala, –ASn, –Cys, –Tyr, –Glu, –Pro, and –Ser (TGA)] were encoded on the light strand.

The phylogenetic tree (Figure 1) was constructed by the maximum-likelihood methods using complete mitochondrial genomes of 17 species from 5 genera. MEGA 7.0 software was used for constructing a maximum likelihood (ML) tree (Kumar et al. 2016). *Pseudecheneis immaculatus* is closely related to *P. sulcata*, which is consistent with the traditional regional classification. Thus, the mitogenome sequence of *P. immaculatus* can contribute to phylogenetic knowledge of the Sisoridae family and provide a useful tool for understanding the genetic diversity, population structure and conservation status of *P. immaculatus* in the future.

**Figure 1.**
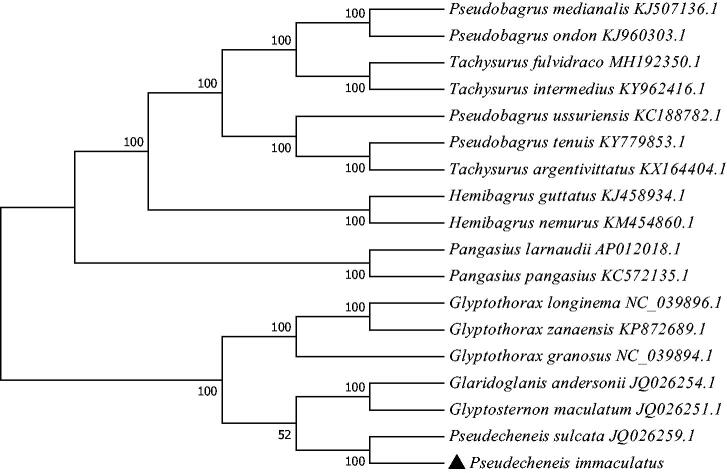
The consensus phylogenetic relationship of the *Pseudecheneis immaculatus* with other fishes. Phylogenetic tree based on the complete mitochondrial genome sequences was constructed by Maximum Likelihood (ML) model. GenBank accession numbers of mitogenomic sequences for each taxon are shown in parentheses.
